# Understanding the transcriptomic response of *Lactiplantibacillus pentosus* LPG1 during Spanish-style green table olive fermentations

**DOI:** 10.3389/fmicb.2023.1264341

**Published:** 2023-09-22

**Authors:** Elio López-García, Antonio Benítez-Cabello, Jordi Tronchoni, Francisco Noé Arroyo-López

**Affiliations:** ^1^Department of Food Biotechnology, Instituto de la Grasa (CSIC), Campus Universitario Pablo de Olavide, Seville, Spain; ^2^Universidad Internacional de Valencia, Comunidad Valencia, Spain

**Keywords:** *Lactobacillus pentosus*, vegetable fermentation, RNA-seq, biofilm, time-course analysis

## Abstract

*Lactiplantibacillus pentosus* (*Lbp. pentosus*) is a species of lactic acid bacteria with a great relevance during the table olive fermentation process, with ability to form non-pathogenic biofilms on olive epidermis. The objective of this work is to deepen into the genetic mechanisms of adaptation of *Lpb. pentosus* LPG1 during Spanish-style green table olive fermentations, as well as to obtain a better understanding of the mechanisms of adherence of this species to the fruit surface. For this purpose, we have carried out a transcriptomic analysis of the differential gene expression of this bacterium during 60 days of fermentation in both brine and biofilms ecosystems. In brines, it was noticed that a total of 235 genes from *Lpb. pentosus* LPG1 were differentially expressed during course of fermentation and grouped into 9 clusters according to time-course analysis. Transport and metabolism of carbohydrates and amino acids, energy production, lactic acid and exopolysaccharide synthesis genes increased their expression in the planktonic cells during course of fermentation. On the other hand, expression of genes associated to stress response, bacteriocin synthesis and membrane protein decreased. A total of 127 genes showed significant differential expression between *Lpb. pentosus* LPG1 planktonic (brine) and sessile (biofilms) cells at the end of fermentation process (60 days). Among the 64 upregulated genes in biofilms, we found genes involved in adhesion (*strA*), exopolysaccharide production (*ywqD, ywqE*, and *wbnH*), cell shape and elongation (*MreB*), and well as prophage excision. Deeping into the genetic bases of beneficial biofilm formation by *Lpb. pentosus* strains with probiotic potential will help to turn this fermented vegetable into a carrier of beneficial microorganisms to the final consumers.

## 1. Introduction

Table olive are one of the most important fermented vegetables in the Mediterranean basin, with an annual worldwide production of 2.8 million tonnes in the 2021/2022 season (International Olive Council, 2023). Lactic acid bacteria (LAB) play a crucial role during the fermentation process of Spanish-style green table olives, the most relevant type of table olive elaboration with approximately 50% of the market. They produce lactic acid after the consumption of sugars, lowering the pH to safe values (<4.2) which prevent the development of undesirable microorganisms and provides product stability (Hurtado et al., 2012). The predominant LAB species involved during the fermentation of lye-treated olives are *Lactiplantibacillus pentosus* (*Lbp. pentosus*) and *Lactiplantibacillus plantarum* (*Lbp. plantarum*; Garrido-Fernandez et al., 1997; Hurtado et al., 2012). In fact, starter cultures containing these species have been developed to ensure a safer food fermentation process (Ruiz-Barba et al., 1994; Rodriguez-Gomez et al., 2014).

Previous studies have demonstrated the ability of LAB species to form non-pathogenic biofilms on olive epidermis including more than 10^7^ CFU/g after concluding fermentation process, albeit they are also present in brine during course of fermentation (10^7^–10^9^ CFU/mL) as planktonic cells (Nychas et al., 2002; Lavermicocca et al., 2005; Arroyo-López et al., 2012; Domínguez-Manzano et al., 2012). There are important differences between the behavior of bacterial cells during its planktonic or sessile state, although both forms are responsible for carrying out the table olive fermentation process. In biofilm, the microorganism are embedded within an extracellular polymeric substance produced by themselves, creating a robust attachment to solid surfaces which confer a great resistance to environmental factors (Davey and O’toole, 2000; Remis et al., 2010). The mechanisms underlying the adhesion of LAB to make these beneficial biofilms are multifaceted, yet they invariably entail a harmonious interplay between the hydrophilic nature of bacterial cell and biotic or abiotic surfaces (Faten et al., 2016). Although essential roles in biofilm formation are played by microbial structures like fimbriae, flagella, and pili, it’s noteworthy that environmental factors, including pH, temperature, exposure duration, and ionic influences, also exert a considerable impact on adhesion processes (Wimpenny, 2009; Domínguez-Manzano et al., 2012; Jahid and Ha, 2012). Biofilms established on the olive surface typically encompass a spectrum of LAB species, prominently *Lbp. pentosus* and *Lbp.* plantarum, alongside diverse yeast species such as *Wickerhamomyces anomalus* and *Saccharomyces cerevisiae*, among others. On the contrary, planktonic cells are designed to colonize new niches, and they have a lower chance of survival to the adverse condition (low pH, high salt concentration, presence of antimicrobial compounds, etc.) usually found during table olive fermentations (Hurtado et al., 2012; Hernández-Jiménez et al., 2013).

*Lbp. pentosus* LPG1 (hereinafter referred to as LPG1) is a microorganism isolated from the biofilms formed during table olive fermentations (Benítez-Cabello et al., 2019). LPG1 has shown remarkable technological features such as esterase and phytase activity, production of lactic acid, bacteriocin production, etc., (Benítez-Cabello et al., 2019). A recent genomic analysis of the LPG1 strain has revealed various genes involved in adhesion, biofilm formation, bacteriocin production, degradation of carbohydrates, and metabolism of phenolic compounds, among these important technological features (López-García et al., 2023a). This microorganism has also shown important potential probiotic features, proving to be an anti-inflammatory agent, reduce cholesterol levels, inhibit foodborne pathogens, and adhere to Caco−2 cells (Benítez-Cabello et al., 2019, 2020). Moreover, LPG1 has recently shown the capacity to modulate the intestinal microbiota of healthy individuals (López-García et al., 2023b).

In a food market dominated mainly by dairy-based probiotic products, the demand for plant-based alternatives has been growing, particularly among vegetarians and individuals with lactose intolerance. In addition to LPG1, other *Lactiplantibacillus* strains isolated from vegetable fermentations have been identified as microorganisms with probiotic potential (Abriouel et al., 2017; Huang et al., 2018; Kim et al., 2020). Thereby, table olives can act as a carrier of beneficial bacteria to consumers. However, it is necessary to deepen in the study of the genes and processes that lead to the formation of beneficial biofilms by *Lpb. pentosus* on olive epidermis.

In this aspect, the use of NGS techniques such as RNA-seq could be very useful in understanding the mechanism involved in biofilm formation. This technique provides a detailed view of the genes expressed at a specific moment. Consequently, it enables the identification and quantification of gene expression levels, facilitating comparisons between planktonic (brine) and sessile (biofilms) populations. Thus, numerous RNA-seq studies have been conducted to investigate the genes responsible for producing the biofilm ecosystem in pathogenic microorganisms (Charlebois et al., 2016; De Celis et al., 2022). However, few transcriptomic studies have focused on *Lpb. pentosus* or *Lpb. plantarum* and the formation of these non-pathogenic biofilms (Alonso García et al., 2021; Zhu et al., 2022). Therefore, an assessment of the genes involved in the biofilm process is necessary, particularly within a plant matrix such as table olive fermentations.

The objective of this study was to evaluate the differential gene expression of LPG1 during table olive fermentation, identifying the genes involved in its adhesion to the olive surface through transcriptomic analysis, mapping against the annotated LPG1 genome.

## 2. Materials and methods

### 2.1. Olive processing

Manzanilla olives were processed according to the Spanish-style during 2021/2022 season in the pilot plant of Instituto de la Grasa (CSIC, Seville, Spain). The fruits were treated with a 2.0% NaOH solution until 2/3 of the pulp was reached. Then, the fruits were washed with tap water for 3 h and placed into 250 mL ISO glass containers (180 gr fruits +136 mL brine). An 11% (w/v) NaCl brine was initially added to cover the olives. Subsequently, the containers were closed and pasteurized at 80°C for 10 min to remove the initial microbial load, and finally stored at 4°C for 7 days until the pH and salt levels were kept constant at 8.1 and 4.9%, respectively. A total of 18 olive containers were used in the present study.

### 2.2. Experimental design

**Figure 1:**
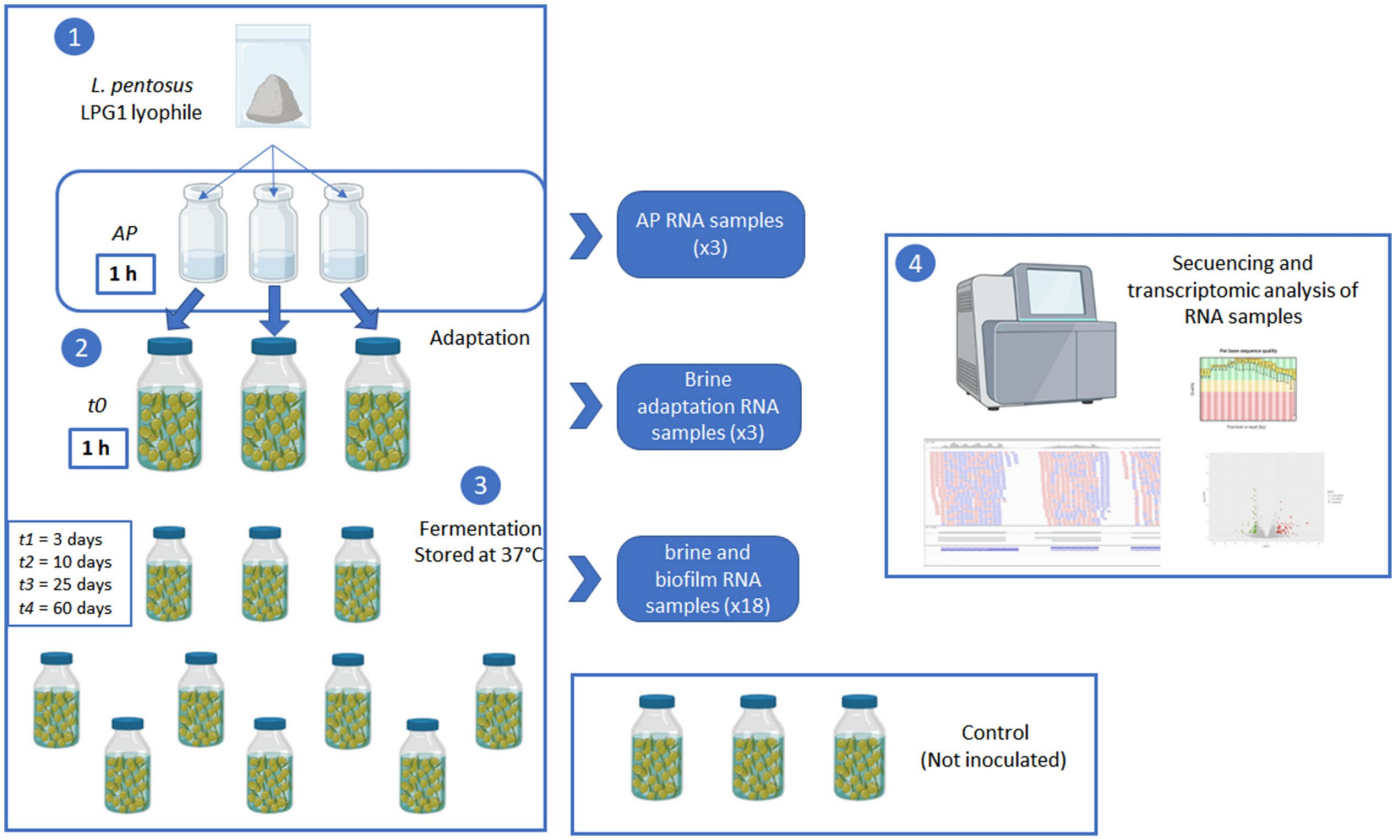
Scheme of the experimental design used in the transcriptomic analysis of LPG1 during Spanish-style green table olive fermentations.

A commercial lyophile of LPG1 (Oleica, Seville, Spain) was reconstituted by triplicate in 0.1% of sterile peptone water (0.1%, w/v) at a concentration of 8 log_10_ CFU/mL. After waiting for 1 h, 5 mL were collected from each individual reconstitution process and referenced as *AP*. Then, 15 pasteurized olive containers were individually inoculated with this reconstituted LPG1 inoculum at a theoretical inoculum concentration of 5 log_10_ CFU/mL. After 1 h, 10 mL of brine were collected from 3 inoculated olive containers and marked as sample *t0*. Then, the rest of inoculated (*n* = 12) and non-inoculated control (*n* = 3) olive containers were stored in the laboratory during 60 days at 37°C. Brine and fruit samples were collected during fermentation with sampling references *t1* = 3 days, *t2* = 10 days, *t3* = 25 days, and *t4* = 60 days. The olive containers were discarded once they were analyzed. All analytical were carried out by triplicate on the individual olive containers. Figure 1 shows a scheme of the experimental design followed in this work.

### 2.3. Physic-chemical and microbiological analysis

At each sampling time, physic-chemical analysis were carried out following the methodology described by Garrido-Fernandez et al. (1997). The NaCl content (%), pH, and titratable acidity (expressed as g of lactic acid per 100 mL of brine) were determined in brine using an automatic titrator model Excellence (Mettler Toledo, Columbus, OH, United States). Samples of brines and fruits were also collected to determine LAB counts using specific MRS agar medium (Oxoid, Basingstoke, Hampshire, United Kingdom) according to methodology described by Benítez-Cabello et al. (2015). Briefly, fruits (10 g) were washed twice with a 0.9% sterile NaCl solution for removing non- and low-adherent cells, pitted, weighed in sterile conditions, and transferred into a stomacher bag containing 25 mL of sterile saline solution. Then, the fruits were homogenized for 3 min at 300 rpm in a stomacher model Seward 400 (Seward, United Kingdom). Decimal dilutions of brines or the stomacher bag liquid were spread onto culture media using a spiral platemaker model easySpiral Dilute (Interscience, Saint Nom la Brétèche, France). The counts were determined using an automatic image analysis system model Scan4000 (Interscience, SaintNom la Brétèche, France) and expressed as log_10_ CFU/mL (brine) or log_10_ CFU/g (biofilm), respectively.

### 2.4. RNA extraction, library preparation and sequencing

For each sampling time point (except *t3*), AP, brine, and biofilm samples were processed for RNA extraction. In the case of olives, prior to centrifugation, the cells adhered to the olive epidermis (non-pathogenic biofilms) were recovered using a stomacher model Seward 400 (Seward, United Kingdom) as described previously. Then, samples were centrifuged at 10.000 rpm during 10 min, and supernatants were carefully removed. In the case of brines, they were also centrifugated in the same conditions described above. Cell pellets were resuspended in 500 ul RNAlater solution and immediately stored at −80°C until further analysis. A total of 24 samples (*AP*-peptone water, *t0*-brine, *t1*-brine, *t1*-biofilm, *t2*-brine, *t2*-biofilm, *t4*-brine, and *t4*-biofilm) were selected for the RNA-seq experiment. Total RNA was extracted using the MasterPure Complete DNA and RNA Purification Kit (LGC Biosearch Technologies, United States) and subjected to DNAase treatment using Baseline Zero DNase (LGC Biosearch Technologies, United States). Once the RNA was extracted from each sample, its integrity was assessed with the Qubit™ RNA IQ kit, and its concentration was measured with the Qubit™ HS RNA kit. Libraries were prepared using the Illumina Stranded Total RNA Prep kit, quantified with the HS DNA Qubit™ kit (Q32851), and then loaded onto the NextSeq500 for sequencing. Sequencing generated 75-bp paired-end sequences. All raw RNA-Seq data have been deposited in ENA repository under PRJEB63117 accession number.

### 2.5. Transcriptomic analysis

Raw reads quality evaluation was performed using the fastp tool (Chen et al., 2018). The mapping of curated reads against the LPG1 genome (accession number PRJEB51357) was carried out using Hisat2 version 2.2.1 (Kim et al., 2015). Alignment parameters included the use of concordant pairs of reads only in the forward-reverse orientation, trimming of 0 bases from the 3′ and 5′ ends, skipping the first *N* reads or pairs in the input as 0, and applying a penalty equal to 1. The quality alignment was measured with Qualimap version 2.2.2d (Okonechnikov et al., 2016). Subsequently, the quantification of mapped transcript in the genome was performed with StrignTie version 2.2.1 (Pertea et al., 2015). The analysis of the differential gene expression was conducted using the Bioconductor package DESeq2 version 1.38.3 in the R statistical software program (Love et al., 2014). A principal component analysis (PCA) was carried out to obtain a broader approach to transcriptomic analysis. Subsequently, the focus was directed to each individual gene. Genes were considered differentially expressed when the Benjamini and Hochberg multiple correction method, commonly known as adjusted value of p or false discovery rate (FDR), was less than 0.05, and the Log_2_ fold change (Log_2_FC) was greater than 2 or less than −2. Moreover, Clusters of Orthologous Genes (COG) enrichment analysis was performed to compare the non-pathogenic biofilm and brine samples.

Due to the exclusion of the samples *t1*-biofilm and *t2*-biofilm (quality control failure), the analysis of differentially expressed genes over time was performed only with brine samples. The time-course analysis was conducted using Bioconductor package maSigPro version 1.70.0 (Conesa et al., 2006). This package fitted a regression model for each gene and returned a list of false discovery rate (FDR) corrected significant genes. An FDR < 0.05 was required to consider a gene as differentially expressed, and the default value 0.6 was used for the regression model R-squared cut-off value.

## 3. Results and discussion

### 3.1. Evolution of fermentation process

**Figure 2:**
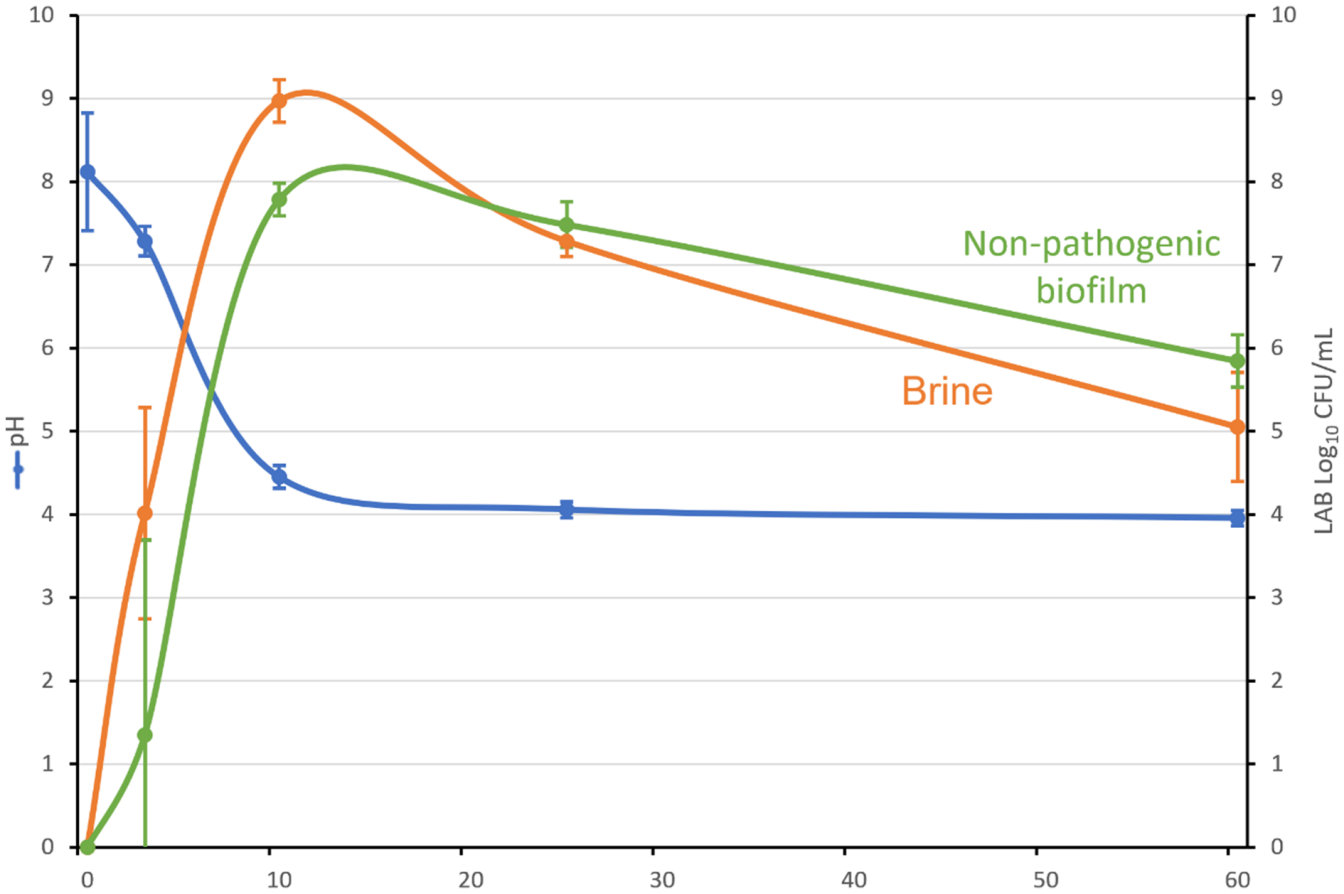
pH evolution, LAB counts in brine (log_10_ CFU/mL) and non-pathogenic biofilms (log_10_ CFU/g) during Spanish-style green table olive fermentation.

All inoculated olive containers followed the usual fermentation process described for this type of table olive elaboration (Garrido-Fernandez et al., 1997). Thereby, the average pH decreased from initial 8.1 to a value close to 4.3 after 10 days of fermentation, and finally reached a value of 4.0 from 25 days onward (Figure 2). The titratable acidity increased from 0.02% at the start of the experiment to final 1.03% due to the fermentation activity of LPG1. On the contrary, the salt levels were kept constant at 4.9% until the end of the experiment. The LPG1 population grew in both brine and biofilm ecosystems, reaching maximum levels in brines at 10 days of fermentation (9 log_10_ CFU/mL) and in non-pathogenic biofilms at 15 days of fermentation (8 log_10_ CFU/g). However, after this moment, the population decreased obtaining an average population of 5.8 log_10_ CFU/g in the mature biofilms at the end of fermentation (60 days), whereas the counts in brine had a higher decline up to 5 log_10_ CFU/mL (Figure 2). Thereby, planktonic LPG1 cells were less resistant to the adverse conditions present during table olive fermentations than those forming biofilms, as described previously by Hernández-Jiménez et al. (2013). In the non-inoculated olive containers, it was not noticed any development of LAB and the pH and salt concentration were kept constant during 60 days of fermentation at 5.64 and 5.1%, respectively. This was indicative that no lactic acid fermentation took place in the control olive containers.

### 3.2. Overview of transcriptomic analysis

In this work, a total of 27 GB raw data were generated by the 24 samples during RNA-seq analysis. Table 1 shows the total raw reads obtained for each sample, the total number of cleaned reads, and the ratio of reads that passed the filter quality. Most of the samples achieved 10 million reads, with some cases exceeding 30 million reads per sample. Thereby, a total of over 400 million reads were obtained, with up to 375 million passing the quality filters. One replicate of *t1*-biofilm and two replicates of *t2*-biofilm lost a large number of reads during quality control, and for this reason, they were removed from the final analysis.

**Table 1:** Reference of samples and total number of reads generated during transcriptomic analysis of LPG1 [before and after quality control (QC) filtering], as well as total number of mapped reads against the LPG1reference genome.

Sample ID	Reads	Reads QC	QC ratio	Reads mapped
*AP*_1	17,175,859	16,821,416	0.979	13,428,895
*AP*_2	11,845,446	11,692,219	0.987	9,215,089
*AP*_3	14,123,905	13,910,956	0.984	11,165,904
*t0*-1	12,264,743	12,117,663	0.988	10,144,415
*t0*-2	13,662,667	13,377,037	0.979	11,271,384
*t0*-3	13,225,408	13,020,146	0.984	11,095,955
*t1*-brine_1	14,131,169	13,923,131	0.985	4,894,826
*t1*-brine_2	10,567,391	10,465,260	0.99	3,528,874
*t1*-brine_3	13,849,491	13,627,733	0.983	11,256,988
*t1*-biofilm_1	17,355,038	16,720,771	0.963	NA
*t1*-biofilm_2	6,822,254	3,725,810	0.546*	NA
*t1*-biofilm_3	31,850,614	31,355,915	0.984	NA
*t2*-brine_1	12,281,519	12,111,635	0.986	9,726,514
*t2*-brine_2	8,258,108	8,100,046	0.98	5,381,781
*t2*-brine_3	12,336,891	12,136,696	0.983	10,630,288
*t2*-biofilm_1	10,636,654	8,883,257	0.835	NA
*t2*-biofilm_2	8,111,896	1,597,390	0.196*	NA
*t2*-biofilm_3	7,913,894	1,218,970	0.154*	NA
*t4*-brine_1	31,829,010	30,514,960	0.958	24,638,646
*t4*-brine_2	32,525,142	31,906,529	0.98	27,255,521
*t4*-brine_3	31,692,228	31,152,365	0.982	24,500,146
*t4*-biofilm_1	21,817,822	21,542,793	0.987	17,637,463
*t4*-biofilm_2	28,013,416	27,617,147	0.985	18,355,990
*t4*-biofilm_3	25,413,662	25,135,624	0.989	17,022,332

^*^Samples with a large loss of readings after quality filtering, which were removed from further analysis. The triplicate of *t1*-biofilm and *t2*-biofilm were not carried out the mapping due to the less quality of some sample (NA).

The LPG1 annotated genome was used for mapping. The genome of LPG1 has a length of 3,700,533 bp distributed among one chromosome and two plasmids, which contain a total of 3,345 protein-coding genes and 89 non-coding sequences (73 tRNA and 16 rRNA genes), with a G + C content of 46.34% (López-García et al., 2023a). Regarding mapping quality, the mean coverage of the samples was more than 300%, and the mean mapping quality of the samples reached a value of 58, with a mean insert size of 154 bp.

**Figure 3:**
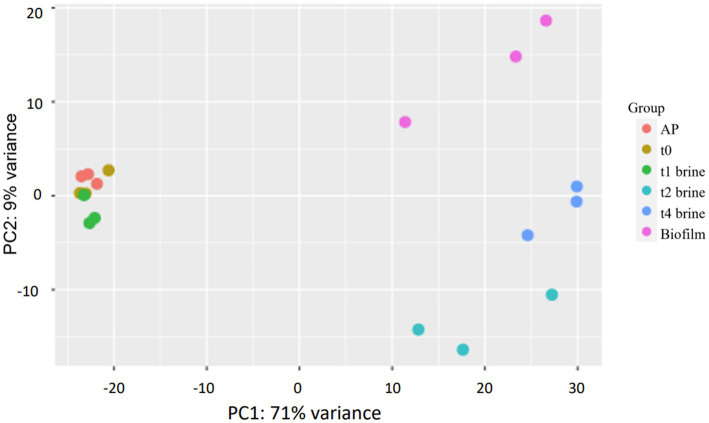
Principal component analysis (PCA) for the 21 samples analyzed which passed quality control. *AP* (lyophiles reconstituted in peptone water 0.1%), *t0* (inoculated samples in brine collected after 1 h), *t1*-brine (brine sample at 3 days of fermentation), *t2-*brine (brine sample at 10 days of fermentation), *t4*-brine (brine sample at 60 days of fermentation), and Biofilm (non-pathogenic biofilm samples at *t4*-60 days of fermentation).

PCA has proved to be a useful statistical tool in numerous RNA-seq studies to initially visualize the clustering of samples in the space. In addition, PCA can help to identify genes responsible for the observed deviations between samples (Wold et al., 1987; Curiel et al., 2017). The first two principal component of the PCA explained a total of the 80% variance of the samples (Figure 3). According to PCA, samples were grouped in 3 regions. The samples from *AP*, *t0*, and *t1*-brine were very similar among them, and they were grouped in the negative part of PC1. In contrast, *t4*-biofilm samples were located in the positive part of PC1 and PC2, while *t2* and *t4*-brine samples were mainly located in the positive part of PC1 and negative part of PC2. Thus, this preliminary analysis clearly reported a differentially gene expression between non-pathogenic biofilm and brine samples at the end of fermentation (*t4*), and between brine samples from beginning (*AP, t0*, and *t1*) and middle-final points (*t2* and *t4*) of fermentation, when low pHs and high titratable acidity levels were obtained. Similar results have been reported in previous studies, where different microbial responses were successfully grouped by means of PCA analysis (Curiel et al., 2017; Zhu et al., 2022).

### 3.3. Short-term adaptation of LPG1 planktonic cells to olive brine

Microorganisms involved in the Spanish-style fermentation process must be capable of fermenting the carbohydrates present in the olive brine (mainly glucose, fructose, saccharose, and mannitol), tolerating a certain salt concentration (6–8% NaCl), growing in the presence of specific phenolic antimicrobial compounds (oleuropein, hydroxytirosol, tyrosol, etc.), and producing a significant amount of lactic acid (Garrido-Fernandez et al., 1997; Medina et al., 2010). As mentioned before, *Lpb. pentosus* and *Lpb. plantarum* are the most abundant LAB species in table olive fermentations (Hurtado et al., 2012). Both species have a remarkable ability to colonize diverse environments due to their enzymatic machinery encoded by relatively large genomes ranging from 3.2 to 4.2 Mb (O’Sullivan et al., 2009; Inglin et al., 2018). Thus, adaptation to the stressing conditions of olive brines is a crucial step for the successful fermentation carried out by *Lpb. pentosus*.

**Figure 4:**
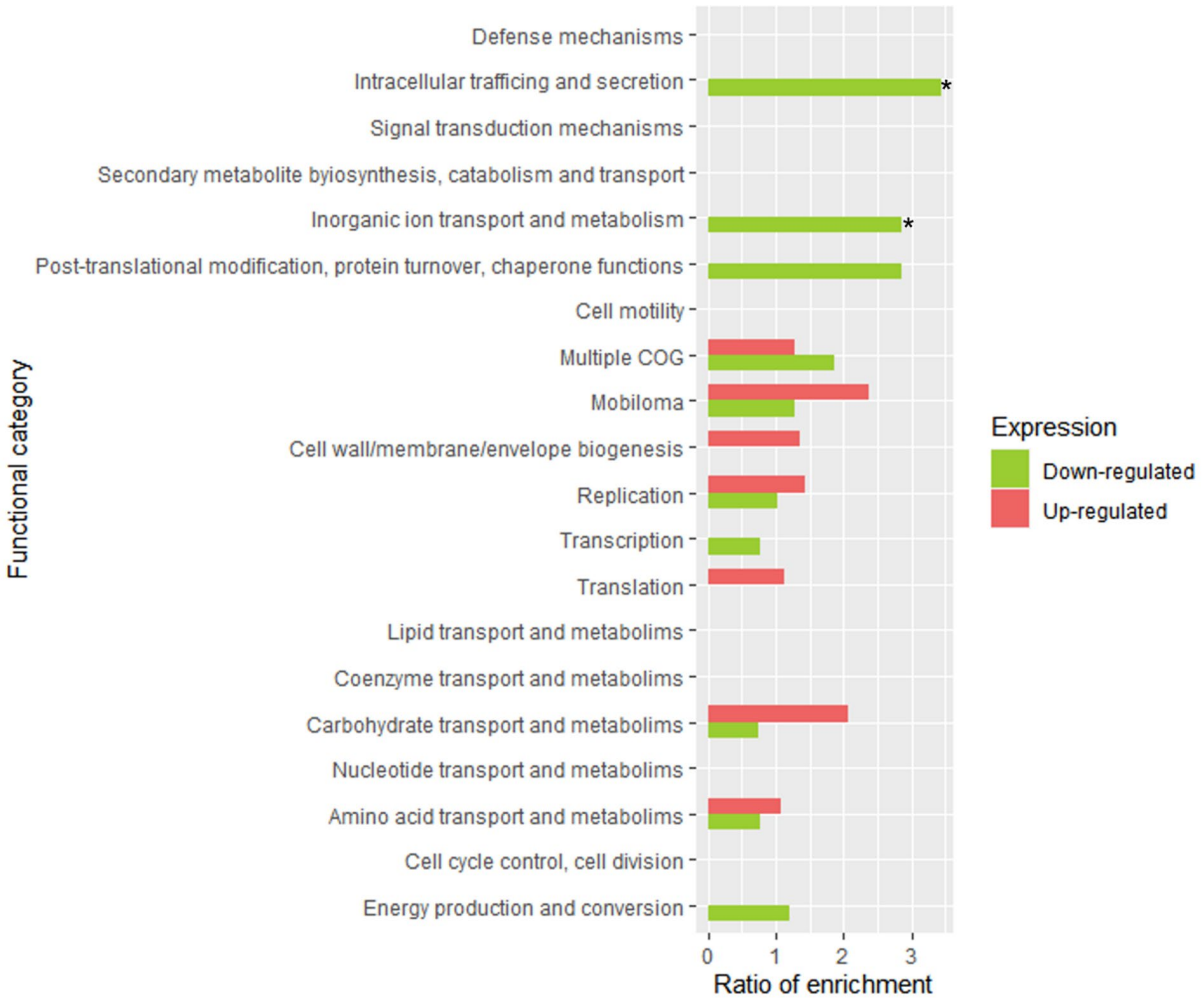
Functional enrichment analysis of clustered genes orthologous (COG) in LPG1 genes in adaptation to brine. The enrichment ratio was calculated as the percentage of genes within a specific functional category in the upregulated or downregulated RNA-seq dataset, relative to the percentage of genes assigned to that functional category in LPG1 genome. * Significant COG categories enrichment amongst genes.

As described above, according to the PCA analysis, no great differences were observed between the samples from *AP* and *t0*-brine. In fact, a differential gene expression analysis yielded a total of 1 upregulated gene and 2 downregulated genes. Therefore, the time elapsed (only 1 h) between the *AP* and *t0-*brine samples was not adequate to observe differentially significant expressions. Thus, *t1-*brine sample (3 days) was chosen to observe the adaptation of LPG1 planktonic cells to olive brine. Figure 4 shows the enrichment of COG categories where the differentially expressed genes were classified. A total of 18 genes were upregulated in *t1*-brine compared to *AP*, while 50 genes were downregulated (Supplementary Table S1). At 3 days of fermentation, LPG1 genes involved in the formation of extracellular exopolysaccharide (EPS) were found upregulated. Moreover, the *LOPJBOPB 01174* gene, encoding the polysaccharide polymerase protein located in an EPS production cluster, was upregulated. This membrane protein, containing up to 10 transmembrane domains, is responsible for polysaccharide polymerization (Deo et al., 2019). Additionally, the *wbbI* gene encoding beta-1,6-galactofuranosyltransferase was also found upregulated. The product of the *wbbI* gene is an enzyme responsible for adding a galactofuranose residue at a specific position of a specific substrate. This addition of the galactofuranose residue contributes to the formation and stability of the bacterial cell wall, as well as resistance to environmental factors (Wing et al., 2006). Therefore, in the early stages of fermentation, the LPG1 planktonic cells increased the expression of genes involved in cellular resilience. It has been described as cell surface modeling acts as an adaptive mechanism for different species in stressful or hostile environments (Martinez et al., 2020). On the other hand, genes involved in sugar transport, amino acid metabolism, and translation were also found upregulated. Although, differential expression of certain genes known to be essential for fermentation adaptation was not observed, numerous transcripts for these genes were detected (Perpetuini et al., 2016). All the genes classified as essential were found to be expressed to some extent. These genes include *eno_1*, *rex*, *mroQ*, *degV_1*, *LOPJBOPB_01576*, *LOPJBOPB_01576*, and *pgi*, which encode for the enolase protein, redox sensing transcriptional repressor Rex, membrane-embedded CAAX protease MroQ, DegV protein, integral membrane protein, transcription regulator TetR, and glucose 6-phosphate isomerase, respectively.

In contrast, the response to oxidative stress was decreased at 3 days of fermentation, as the genes *msrB* (peptide methionine sulfoxide reductase MrsB), *garB_2* (glutathione amide reductase), and *perR* (peroxide operon regulator) were downregulated. These gene products are related to the response to oxidative stress. Glutathione amide reductase helps to maintain the redox balance in cells by participating in the regeneration of reduced glutathione, which is an important antioxidant molecule involved in detoxification processes and protection against oxidative stress (Pophaly et al., 2012). MrsB protein reduces methionine sulfoxide residues (oxidized methionine) in proteins, restoring their functionality (Grimaud et al., 2001). Finally, the protein encoded by *perR* gene acts as a transcriptional repressor that controls the expression of genes involved in the response to oxidative stress (Mongkolsuk and Helmann, 2002). Thereby, all these downregulated proteins prevent the accumulation of oxidative compound in the cell and contribute to cellular redox homeostasis. Downregulation of these genes may be due to the low oxidative stress and the cell repressing this expression to conserve energy. Similarly, genes encoding proteins involved in resistance to thermic, oxidative, osmotic, or toxic stress, such as *LOPJBOPB 03141* (belongs to the small heat shock protein (HSP20) family), *spxA* (regulatory protein Spx), *gla* (glycerol facilitator-aquaporin gla), and *LOPJBOPB 03204* (belongs to the dps family), were found to be downregulated. The results obtained show that after 3 days of fermentation, the cellular stress of the LPG1 strain is lower than after its reconstitution in peptone water, as numerous stress-responsive genes were found to be downregulated, which is indicative that the bacteria is already well adapted to the olive fermentation conditions.

### 3.4. LPG1 planktonic cell gene expression during fermentation

**Figure 5:**
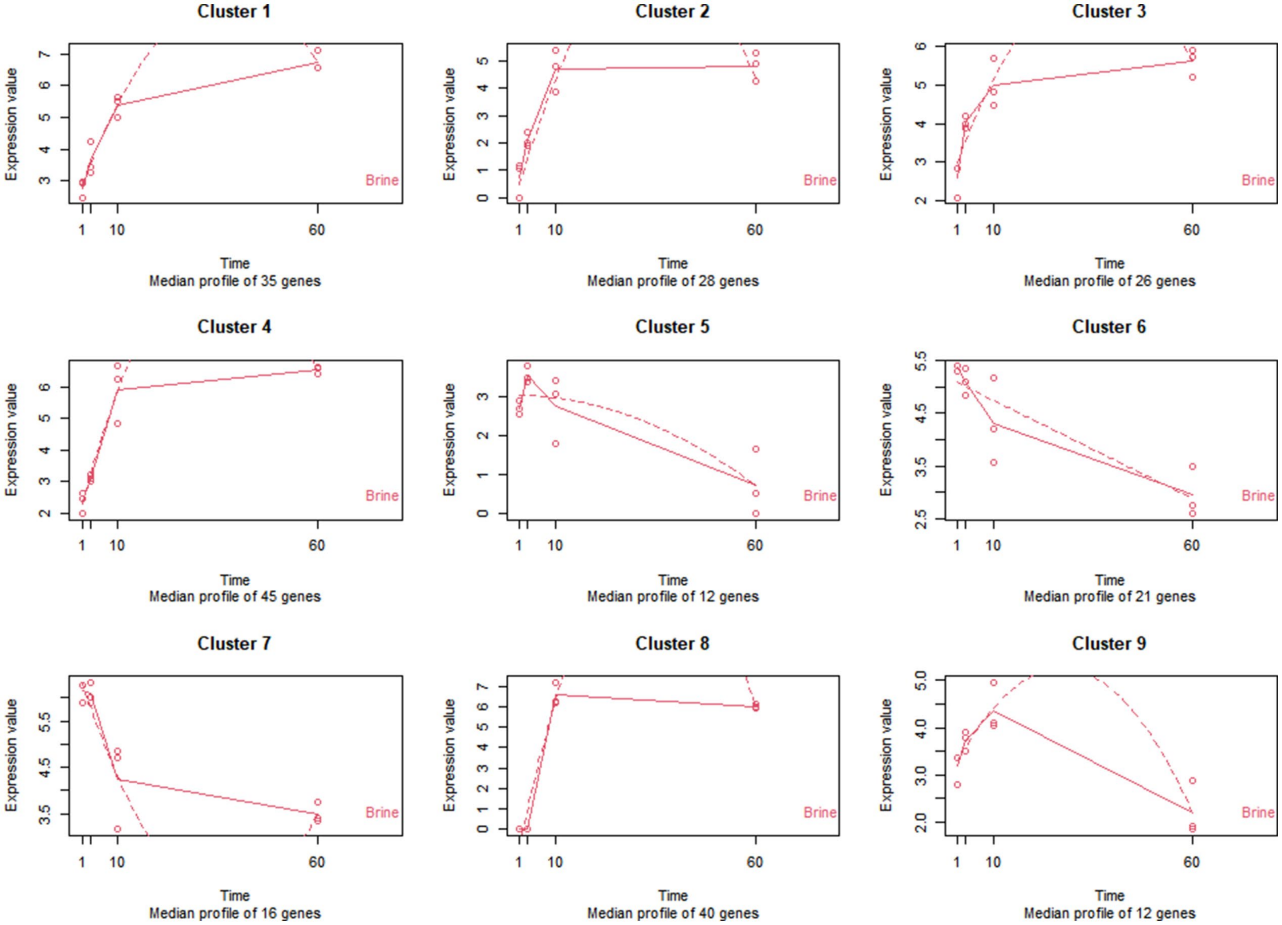
Patterns of expression of the 9 clusters of genes identified in the time-course analysis of LPG1 strain in brine. The expression values are expressed in TPM (transcripts per million). The solid line represents the actual values, and the dashed line represents the regression curve.

Throughout 60 days of fermentation, a total of 235 genes from LPG1 planktonic cells were significantly differentially expressed in brine (Supplementary Table S2). These genes were grouped into 9 clusters according to time-course analysis (Figure 5).

Genes grouped in clusters 1 to 4 had an increase in expression during 60 days of fermentation. Transport and metabolism of carbohydrates and amino acids, energy production, lactic acid and EPS synthesis were the prominent functions observed in gene clusters 1 to 4. The exponential growth of the LPG1 population in brine could be mainly due to the utilization of a wide variety of sugars present in the brine, such as sucrose, glucose, fructose, and mannitol, and the ability of the LPG1 strain to hydrolyze them. According to the time-course analysis, cluster 4 exhibited higher gene expression than the other clusters at 10 days. In cluster 1 and 4, genes encoding key enzymes were found for sucrose hydrolysis, such as oligo-1,6-glucosidase and sucrose-6-phosphate hydrolase, respectively. Similar results have been observed in the carbohydrate metabolism of *Lpb. pentosus* during adaptation in olive oil (Alonso García et al., 2021). Additionally, gene encoding the enzyme mannitol-1-phosphate 5-dehydrogenase responsible for mannitol hydrolysis was also found in this cluster. Furthermore, the gene encoding mannitol-specific phosphotransferase enzyme IIA component, involved in mannitol transport into the cell, was present in cluster 4. Also present in this cluster was the gene encoding the fructokinase enzyme responsible for the phosphorylation of fructose for subsequent utilization in glycolysis. On the other hand, cluster 1 showed higher expression than cluster 4 at the end of fermentation. Within cluster 1, several genes involved in glucose metabolism were found, including *gdh* encoding glucose 1-dehydrogenase, which catalyzes the oxidation of glucose to glucose-6-phosphate. This glucose-6-phosphate can be subsequently utilized for energy production through glycolysis. Additionally, the *glcU* gene (glucose uptake protein), responsible for transporting glucose across the membrane, was also present in cluster 1. Several genes involved in carbohydrate metabolism were found in these 4 clusters, more specifically in galactose metabolism, such as genes implicated in synthesis (*galT*), transport (*lacS*, *agaC*, and *gatC*) and degradation (*lacL* and *lacM*). The presence of galactose, which is a sugar not commonly associated with the fermentation of table olives, suggests that LPG1 strain could has the ability to produce this carbohydrate. In fact, within these four clusters, various genes were found to be differentially expressed over time, indicating their involvement in carbohydrate biosynthesis and extracellular translocation. Specifically, genes such as *glgE* (Alpha-1,4-glucan:maltose-1-phosphate maltosyltransferase), *fba* (Fructose-bisphosphate aldolase), *LOPJBOPB_01767*, *galT* (galactose-1-phosphate uridylyltransferase), and *ppsA* (phosphoenolpyruvate synthase) were implicated in anabolic processes related to carbohydrate metabolism. Conversely, *LOPJBOPB_02006* (oligosaccharide flippase family protein) gene was associated with the transport of carbohydrates to the extracellular space, contributing to the formation of the EPS. EPS, composed of carbohydrates, proteins, nucleic acid, and lipid, primarily acts as a protective barrier against external agents. Moreover, EPS plays a critical role in biofilm formation and maturation (Dertli et al., 2015). However, under nutrient-limited conditions, bacteria can use as fermentable substrate the EPS previously produced (Rios-Covian et al., 2016). It is noteworthy that lactic acid production carried out by *ldhD* (D-lactate dehydrogenase) gene included in cluster 2 is essential for lowering pH during fermentation.

On the contrary, genes of cluster 5 showed a pattern of increase their expression up to 3 days and then decrease until the end of fermentation. Within this cluster, *dps* gene encoding a DNA protection during starvation protein stands out. The function of this protein is to protect and preserve DNA integrity during adverse conditions. Therefore, when LPG1 was under unfavorable conditions the expression of the *dps* gene increased and when the pH started to decrease in brine the expression of the *dps* gene decreased. Previous studies have shown the rapid degradation of the protein encoded by the *dps* gene during the exponential growth phase (Stephani et al., 2003). In addition, the *lexA* gene coding for the LexA repressor maintained the expression of cluster 5. This repressor is responsible for inhibiting the SOS response network that activates in response to DNA damage. However, LexA repressor also affects the expression of genes involved in other functions. In previous transcriptomic studies, the *lexA* gene was found to be downregulated during biofilm formation process (Philips et al., 2017).

Genes of clusters 6 and 7 displayed a decrease expression over time. The primary functions associated with genes of clusters 6 and 7 were stress response, bacteriocin synthesis and membrane protein expression. Up to two annotated bacteriocin immunity genes were downregulated over time as shown the cluster 6, and a bacteriocin gene called *LOPJBOPB 00365* was located into plantaricin bacteriocin cluster. Previous studies of bacteriocin production modeling reported an increase in bacteriocin production during exponential cell growth. Furthermore, after 50 h of cultivation, bacteriocin production decreased, which is consistent with the behavior observed for LPG1 in brine (Zhou et al., 2015). On the other hand, *perR* gene which encodes to peroxide operon regulator was also included in cluster 6. When LPG1 faces oxidative stress, such as the onset of fermentation, the peroxide operon increased its expression. Thus, as fermentation progresses, the oxidative stress decreases and consequently the expression of *perR.* Most of the genes included in cluster 7 were assigned to hypothetical proteins, after a homology search, the proteins encoded by these genes were identified as cell surface proteins. Cell surface remodeling occurs through the plasticity of the cell wall, and the expression of various surface proteins is a common phenomenon throughout the cellular life, primarily influenced by environmental conditions and the specific niche (Martinez et al., 2020).

Finally, genes from clusters 8 and 9 increased their expression up to 10 days and subsequently experienced a decrease throughout the fermentation process, with cluster 8 showing only a slight decrease. Genes involved in the transport and metabolism of amino acids, as well as those related to transcriptional regulation and membrane protein expression, were classified within these clusters.

### 3.5. Comparison of gene expression between sessile and planktonic LPG1 cells at the end of fermentation

During the fermentation of table olives, LAB exhibit progressively a strong adherence to the olive skin, forming a non-pathogenic biofilm on the fruit. This holds great importance given the probiotic potential presented by this specific strain of *Lpb. pentosus* (Benítez-Cabello et al., 2019, 2020; López-García et al., 2023b). Therefore, LPG1 needs to express genes encoding surface adhesion proteins, as observed in previous studies on diverse *Lpb. plantarum* and *Lpb. pentosus* strains (Ao et al., 2020; Sun et al., 2020). A total of 127 genes showed significant differential expression between non-pathogenic biofilms (sessile) and brines (planktonic cells) at the end of fermentation process (*t4*–60 days), with 64 genes upregulated and 63 genes downregulated, using the aforementioned threshold (Supplementary Table S3). Surprisingly, none of these genes were related to the quorum sensing phenomena described previously for diverse LAB species, with no changes in the levels of expression of histidine protein kinases or *luxS* genes (Man and Xiang, 2021).

**Figure 6:**
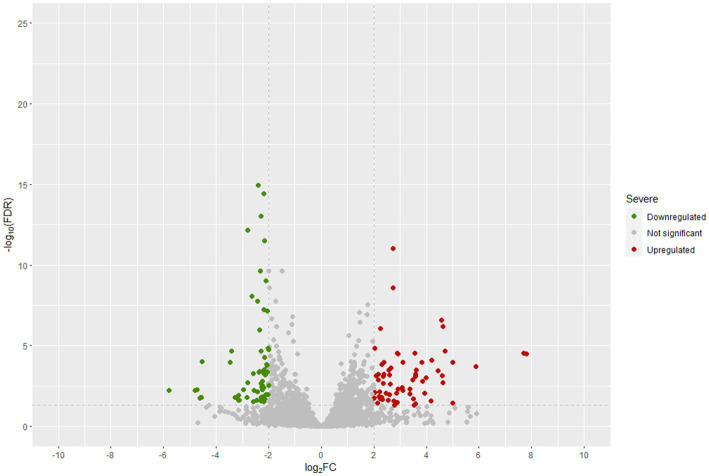
Differentially expressed genes (DEGs) in *t4*-biofilm samples compared to *t4*-brines samples at 60 days of fermentation.

**Figure 7:**
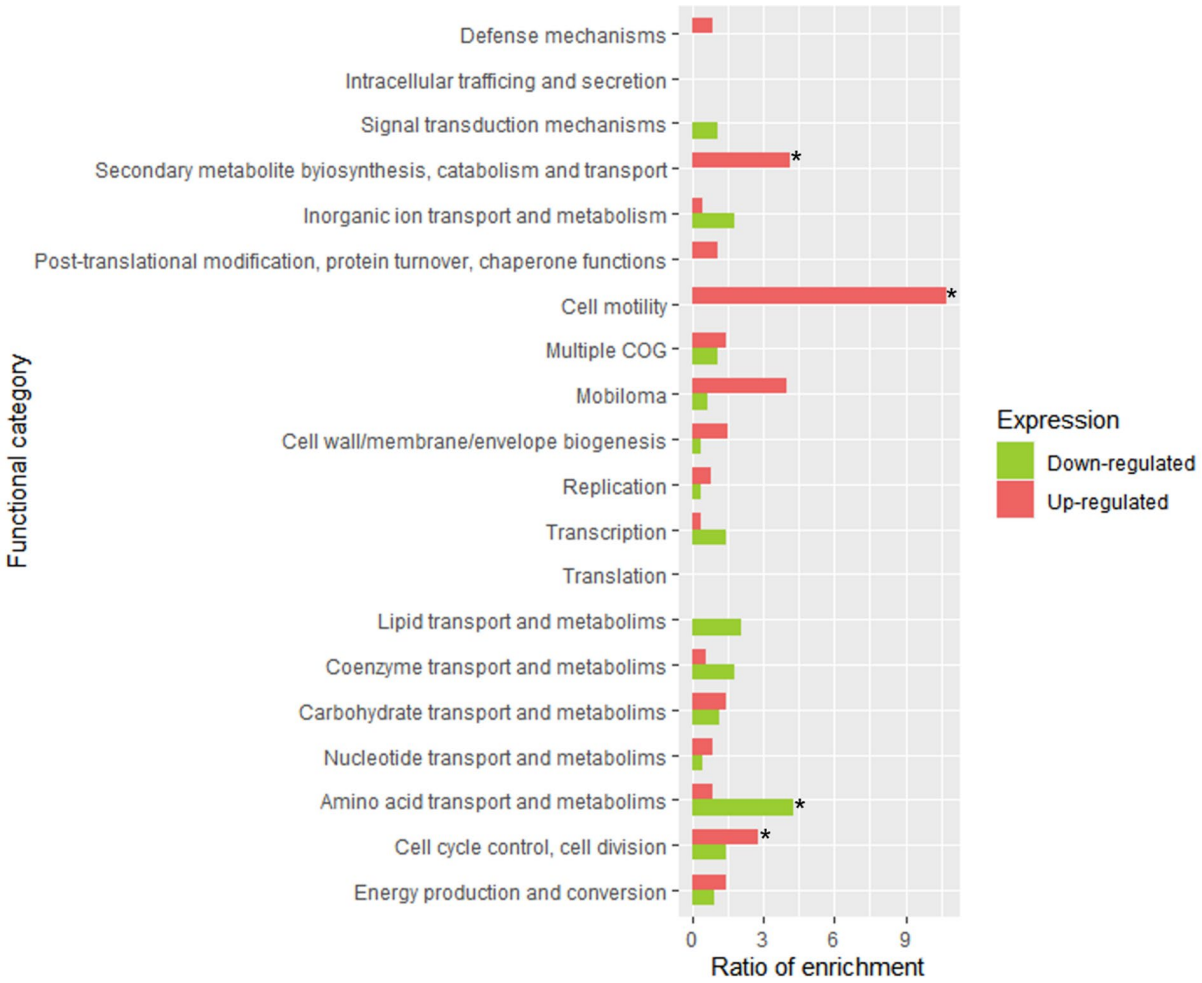
Functional enrichment analysis of clustered genes orthologous (COG) in LPG1 non-pathogenic biofilm (*t4*-biofilm) at 60 days of fermentation. The enrichment ratio was calculated as the percentage of genes within a specific functional category in the upregulated or downregulated RNA-seq dataset, relative to the percentage of genes assigned to that functional category in LPG1 genome. * Significant COG categories enrichment amongst genes.

Figure 6 displays the statistical significance (− Log_10_ FDR) and fold change (Log_2_ FC) of the analyzed genes. The number of significantly differentially expressed genes was lower than in other transcriptomic studies of *Lpb. plantarum* and *Lpb. pentosus* (Ao et al., 2020; Sun et al., 2020; Alonso García et al., 2021). However, this was likely due to the highly restrictive thresholds used in the present study, as the decision was made to consider genes that were four times more/less expressed (Log_2_FC = |2|). This approach allowed for a more confident assumption of differential expression. Enrichment analysis of COG categories was conducted to compare the enriched functional categories in the non-pathogenic biofilm and brine of LPG1 populations (Figure 7). Up to 4 COG categories were significantly enriched in non-pathogenic biofilms. The categories of cell motility, secondary metabolite biosynthesis, catabolism/transport, and cell cycle control/division were significantly enriched due to the upregulated genes in these categories. Conversely, the amino acid transport and metabolism category was significantly enriched due to the downregulated genes within this category.

Table 2 summarized the genes included in the four significantly enriched COG categories. Among the upregulated genes in sessile cells, several genes involved in adhesion were identified based on the literature. The *strA* gene encoding sortase A protein, which has been associated with adhesion to epithelial cells, exhibited significant upregulation in the non-pathogenic biofilm samples, indicating its key role in adhesion to the surface of fruit during the fermentation process by LPG1 strain (Lalioui et al., 2005). Additionally, genes involved in non-pathogenic biofilm formation were detected, including three upregulated genes belonging to an EPS production cluster: *ywqD* (tyrosine-protein kinase), *ywqE* (tyrosine-protein phosphatase), and *wbnH* (O-antigen biosynthesis glycosyltransferase). EPS plays a fundamental role in the formation, stability, and functionality of the biofilm, and a decrease in EPS production resulted in reduced biofilm formation in *Pseudomonas aeruginosa* (De Celis et al., 2022). On the other hand, the *lrgB* gene, encoding the Antiholin-like protein LrgB, is part of the well-known LrgAB system into *lrg* operon. The *lrgA* and *lrgB* genes are annotated in the database as a regulator and effector of murein hydrolase, respectively. Previous studies have shown that mutations in the LrgAB system affect various cellular functions, including autolysis, biofilm formation, and response to oxidative stress (Ahn et al., 2010). *Streptococcus mutans lrgB* mutants exhibited decreased biofilm-forming capacity and increased autolysis compared to the wild-type strain (Rice et al., 2017). However, *Bacillus cereus* and *Staphylococcus aureus lrgB* mutants showed increased biofilm formation compared to the wild-type strain. The release of extracellular genomic DNA (eDNA) through cell lysis by *lrgB* mutant strains promoted biofilm development (Mann et al., 2009; Zhang et al., 2020). Thus, the overexpression of *lrgB* in LPG1 may act by promoting autolysis and enhancing the stability and cohesion of the biofilm through eDNA.

**Table 2:** Summary of the genes included in the four significantly enriched COG categories obtained during differential gene expression between sessile and planktonic LPG1 cells at the end of fermentation (60 days).

Gene_ID	Log2_ fold_change	*q*-value	Gene_name	Function	COG category
*LOPJBOPB_02959*	2,309,984,228	0.00014	*coq5*	2-Methoxy-6-polyprenyl-1,4-benzoquinol methylase/Methyltransferase domain	Secondary metabolites biosynthesis, transport and catabolism
*LOPJBOPB_01282*	2,629,517,511	0.00234	*LOPJBOPB_01282*	Cell shape-determining protein MreB	Cell motility
*LOPJBOPB_00230*	−2,396,389,007	0	*cysE*	Serine acetyltransferase	Amino acid transport and metabolism
*LOPJBOPB_00231*	−2,107,295,766	0	*metB*	Cystathionine gamma-synthase	Amino acid transport and metabolism
*LOPJBOPB_00232*	−2,305,432,622	0	*cysM*	Cysteine synthase	Amino acid transport and metabolism
*LOPJBOPB_00328*	−2,795,452,187	0	*bacF*	Transaminase BacF	Amino acid transport and metabolism
*LOPJBOPB_00385*	−2,421,998,712	0	*sarA_1*	Oligopeptide-binding protein SarA	Amino acid transport and metabolism
*LOPJBOPB_01056*	−2,003061072	0.00303	*aroE_1*	Shikimate dehydrogenase (NADP(+))	Amino acid transport and metabolism
*LOPJBOPB_01057*	−2,139,802,071	0.00028	*aroF*	Phospho-2-dehydro-3-deoxyheptonate aldolase	Amino acid transport and metabolism
*LOPJBOPB_01058*	−2,1,871,927	0.00052	*aroB*	3-Dehydroquinate synthase	Amino acid transport and metabolism
*LOPJBOPB_01953*	−2,247,627,779	0.00307	*aroC*	Chorismate synthase	Amino acid transport and metabolism
*LOPJBOPB_02430*	−2,243,050,584	0.00283	*hisC*	Histidinol-phosphate aminotransferase subfamily	Amino acid transport and metabolism
*LOPJBOPB_02436*	−4,619,788,071	0.01727	*hisB*	Imidazoleglycerol-phosphate dehydratase	Amino acid transport and metabolism
*LOPJBOPB_03265*	−2,206,948,069	0.00358	*aroE_3*	Shikimate dehydrogenase (NADP(+))	Amino acid transport and metabolism
*LOPJBOPB_03266*	−2,298,662,217	0.00219	*aroE_4*	Shikimate dehydrogenase (NADP(+))	Amino acid transport and metabolism
*LOPJBOPB_01168*	5,8,886,018	0.00018	*ywqD_1*	Tyrosine-protein kinase	Cell cycle control, cell division
*LOPJBOPB_03161*	2,38,681,669	0.00059	*menH_7*	2-Succinyl-6-hydroxy-2,4-cyclohexadiene-1-carboxylate synthase	Cell cycle control, cell division

In other way, the upregulated gene associated with the enrichment of the motility COG category was *LOPJBOPB_01282*, encoding the cell shape-determining protein MreB. The MreB protein acts by regulating cell wall synthesis and elongation, thereby influencing cell shape. However, MreB protein has also been implicated in chromosomal segregation and cell polarity (Shih and Rothfield, 2006). Inhibition or loss-of-function mutations in the MreB protein result in cells adopting a spherical shape. *Rhodobacter sphaeroides* and *Pseudomonas aeruginosa* with inhibited MreB protein exhibited reduced ability to form biofilms. The authors suggested that cell shape affects biofilm formation by reducing the available surface for bacterial adhesion (Lin et al., 2015; Bonez et al., 2017). However, MreB protein also plays a role in oxidative stress response, as observed in *Vibrio parahemolyticus* and *Latilactobacillus sakei* (Chiu et al., 2008; Chiaramonte et al., 2010). Thus, overexpression of the *mreB* gene in LPG1 in mature non-pathogenic biofilms can have consequences on the structure and functionality of the biofilm. Finally, it is worth noting the overexpression of 21 genes belonging to a prophage out of a total of 58 genes comprising it. Considering the proportion of upregulated genes in the prophage relative to the total number of upregulated genes in the genome, the overexpression of prophage was found to be statistically significant. A prophage is the life form of a bacteriophage (bacterial virus) during its lysogenic cycle, where the phage incorporates into the bacterial genome (Moineau, 2013). Genes encoding major and minor capsid proteins, tail protein, head and tail connector protein, small and large subunits of the terminase were found to be upregulated in the biofilm. Previous RNA-seq studies have revealed that *Vibrio parahaemolyticus*, after the removal of prophage, exhibited a reduced ability to form biofilms due to a change in the cell surface hydrophobicity (Xu et al., 2022). However, few studies have been conducted on prophage induction within the biofilm context. Most studies have focuses on prophage release after bacterial death, where the liberated prophage can infect other bacteria. Therefore, further studies are needed to elucidate the role of prophages in the non-pathogenic biofilm formation and stability of *Lpb. pentosus*.

On the contrary, the genes downregulated in the non-pathogenic biofilm respect to brines were associated in a large number with amino acid transport and metabolism, as well as transcriptional regulations. Genes such as *cysE*, *metB*, and *cysM* were found to be downregulated and are clustered together. This gene cluster is involved in the synthesis of cysteine and methionine, which are essential amino acids for protein synthesis and cellular function. The proximity of these genes within the bacterial genome suggests coordinated regulation of their expression (Wüthrich et al., 2018).

Genes including *aroE_1*, *aroE_3*, *aroE_4*, *aroF*, *aroB*, *LOPJBOPB_01952*, and *aroC*, which are involved in the metabolism of shikimic acid were downregulated. The shikimate pathway plays a crucial role in the synthesis of aromatic amino acids such as phenylalanine, tyrosine, and tryptophan, as well as vitamins, folic acid, and other metabolites (Panina et al., 2003). Furthermore, genes *hisC* and *hisB*, responsible for histidine synthesis, were also downregulated. The downregulated genes in non-pathogenic biofilm involved in amino acid metabolism and transport has been previously reported (De Angelis et al., 2015; Liu et al., 2022). Zhao et al. (2021) speculated that the downregulated genes involved to amino acid synthesis pathways could be due to the presence of high levels of NaCl.

In contrast to bacterial cells within the non-pathogenic biofilm, planktonic cells in brines exhibited an overexpression of the *lrgA_1* gene, encoding the Antiholin-like protein LrgA. This gene was predicted to encode the LrgA protein included in the aforementioned LrgAB system. As seen, the LrgAB system is involved in various cellular functions. As LrgB protein, LrgA protein is engaged with autolysis activity and biofilm formation. In this case, the LrgA protein encodes the regulator of murein hydrolase. Previous studies with *S. mutans lrgA* mutants have shown increased autolysis compared to lrgB mutants. Additionally, the product of the *lrgB* gene has been suggested to play a more significant role in stress tolerance compared to the *lrgA* gene (Ahn et al., 2010). Therefore, the *lrgA* gene does not appear to be essential for non-pathogenic biofilm formation by LPG1 strain.

## 4. Conclusion

To our knowledge, this is the first work on the global gene expression of a *Lpb. pentosus* strain during table olive fermentations. LPG1 showed a differential gene expression in brine during course fermentation, but also, between planktonic and sessile cells at the end of fermentation process. Deeping into the genetic bases of non-pathogenic biofilm formation by *Lpb. pentosus* strains with probiotic potential will help to turn this fermented vegetable into a carrier of beneficial microorganisms to the final consumers as an alternative to dairy products.

## Data availability statement

The datasets presented in this study can be found in online repositories. The names of the repository/repositories and accession number(s) can be found at: https://www.ebi.ac.uk/ena, PRJEB63117.

## Author contributions

EL-G: Formal analysis, Methodology, Writing – original draft, Writing – review & editing, Data curation. AB-C: Formal analysis, Methodology, Writing – original draft, Writing – review & editing. JT: Formal analysis, Supervision, Writing – review & editing. FA-L: Conceptualization, Funding acquisition, Project administration, Supervision, Writing – original draft, Writing – review & editing.
